# Phylogeographic Insights into a Peripheral Refugium: The Importance of Cumulative Effect of Glaciation on the Genetic Structure of Two Endemic Plants

**DOI:** 10.1371/journal.pone.0166983

**Published:** 2016-11-21

**Authors:** Gabriele Casazza, Fabrizio Grassi, Giovanni Zecca, Luigi Minuto

**Affiliations:** 1 DISTAV, Università degli studi di Genova, Genova, Italy; 2 Dipartimento di Biologia, Università degli studi di Bari, Bari, Italy; 3 Dipartimento di Bioscienze, Università degli studi di Milano, Milano, Italy; Universita degli Studi di Milano-Bicocca, ITALY

## Abstract

Quaternary glaciations and mostly last glacial maximum have shaped the contemporary distribution of many species in the Alps. However, in the Maritime and Ligurian Alps a more complex picture is suggested by the presence of many Tertiary paleoendemisms and by the divergence time between lineages in one endemic species predating the Late Pleistocene glaciation. The low number of endemic species studied limits the understanding of the processes that took place within this region. We used species distribution models and phylogeographical methods to infer glacial refugia and to reconstruct the phylogeographical pattern of *Silene cordifolia* All. and *Viola argenteria* Moraldo & Forneris. The predicted suitable area for last glacial maximum roughly fitted current known distribution. Our results suggest that separation of the major clades predates the last glacial maximum and the following repeated glacial and interglacial periods probably drove differentiations. The complex phylogeographical pattern observed in the study species suggests that both populations and genotypes extinction was minimal during the last glacial maximum, probably due to the low impact of glaciations and to topographic complexity in this area. This study underlines the importance of cumulative effect of previous glacial cycles in shaping the genetic structure of plant species in Maritime and Ligurian Alps, as expected for a Mediterranean mountain region more than for an Alpine region.

## Introduction

Quaternary glaciations and mostly late Pleistocene glaciation (0.120–0.018 Ma) have shaped the contemporary distribution of many species [1]. In general, species survived cold adverse periods in so-called glacial refugia and later recolonised areas newly available during warmer post-glacial periods [2, 3]. Populations that survived glaciations in refugia could have accumulated large amounts of genetic diversity, whereas those now found in post-glacially recolonised regions may have less diversity as a result of bottlenecks during their recent expansions [3, 4].

In the last few years several phylogeographical studies have supported this general view concerning the importance of the last glacial maximum (LGM; about 0.020 Ma) in shaping species distribution [5] and contemporary intraspecific genetic diversity [6, 4]. However, it has also been recently demonstrated that Mediterranean refugia represent climatically stable areas for which it is necessary to consider the cumulative effects of changes, rather than just the changes that occurred during the last glacial period [7]. The high genetic diversity detected in the Mediterranean refugia is due to the preservation of genotypes that went extinct in other places and to the intensity and accumulation of a number of processes in a patchy landscape across a varied topography [8]. A detailed understanding of the effects of past climate changes on the distribution and genetic pattern of organisms may help us to better predict the effects of ongoing climate change [9, 10]. It has been recently demonstrated that the combination of paleo-distribution modelling with phylogeographical approaches may led to new interpretations of population genetic patterns and to new hypotheses about glacial survival and postglacial colonization [11, 12, 13].

Situated at the crossroads of the Mediterranean Basin and the Alps, the Maritime and Ligurian Alps are a hotspot for plant biodiversity [14, 15] and well-known peripheral refugia [16]. Molecular investigations on the endemic plants of this region have underlined the role of vicariance events in modelling the genetic pattern of species during the last glaciation [17, 18, 19]. Nevertheless, Casazza et al. [13] recently demonstrated that the narrow endemic *Primula allionii* Loisel. was separated into two geographically disjoined groups during the Early/Mid Pleistocene border (0.781 Ma) and the region probably contains other paleoendemic species representing living examples of an ancient Tertiary flora present prior to the onset of the Mediterranean climate regime and subsequent Quaternary glaciations. However, the low number of endemic species that have been studied in this region limits our capacity to understand the processes that took place within the Maritime and Ligurian Alps [13, 17, 18, 19, 20].

**Fig 1:**
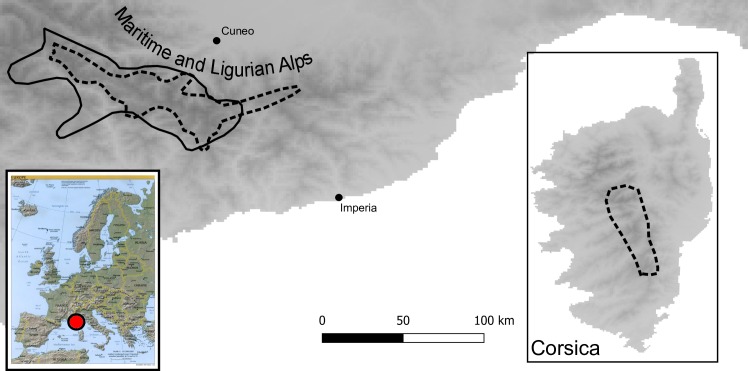
Area of distribution of *S*. *cordifolia* (continuous black line) and *V*. *argenteria* dotted black line). The names of major geographical areas are reported.

In the present study we used species distribution models (SDMs) and phylogeographical methods to infer glacial refugia and to reconstruct the phylogeographical pattern of two endemic species of the area, *Silene cordifolia* All. and *Viola argenteria* Moraldo & Forneris (Fig 1). *Silene cordifolia* is a strict endemic species restricted to the Maritime Alps that represents an old Tertiary lineage [21] and currently occurs on siliceous cliffs and erratic rocks between 700 and 3000 m of altitude. *Viola argenteria* is a disjoint endemic species (*sensu* [22]) that has a small number of populations on Corsica (Fig 1). This species grows on scree slopes from 2000 to 3000 m altitude and may have originated more recently, as suggested by morphological, cytogenetic and molecular evidences [23]. More specifically, we asked the following questions: (1) Is there any evidence of haplotype divergence within *S*. *cordifolia* and *V*. *argenteria* before the last Pleistocene glaciation? (2) Is there any evidence that populations of *S*. *cordifolia* and *V*. *argenteria* were influenced by Pleistocene climate fluctuations, and, if so, how?

## Materials and Methods

### Species distribution modelling

We collected all occurrence data from the SILENE database (www.silene.eu) of the Conservatoire Botanique National Méditerranéen de Porquerolles (CBNMed), from Florence, Turin and Genoa Herbaria (FI, TO, GE) and from our own field surveys. We thus gathered a total of 369 points (266 from SILENE, 49 from Herbaria and 54 from field surveys) for *S*. *cordifolia* and 224 points (121 from SILENE, 54 from Herbaria and 49 from field surveys) for *V*. *argenteria*.

At the local scale, species distribution models provide excellent spatial projections by additionally using non-climatic predictors [24]. For this reason, a layer reporting the presence/absence of suitable substrate was elaborated, starting from the global lithological map dataset, GLiM [25]. The bioclimatic layers for the current (30 arcsec resolution) and LGM (2.5 arcmin resolution) conditions, data were downloaded from the WorldClim 1.4 database website (http://www.worldclim.org). We used data from the Community Climate System Model (CCSM), the Model for Interdisciplinary Research on Climate (MIROC) and from the Max Planck Institute for Meteorology (MPI) to hindcast LGM (http://www.worldclim.org/paleo-climate1#com). These data were statistically downscaled from the original resolution to 30 arcsec with the Delta method [26]. This downscaling method is based on the interpolation of anomalies (differences between LGM and current conditions) at 30 arcsec resolution through the thin-plate smoothing spline algorithm. The interpolated anomalies were finally added to the layers of current condition. To reduce the multicollinearity between predictors and to minimize model overfitting, after normalizing the 19 bioclimatic predictors we performed a pairwise Pearson correlation between them (S1 Table) using R v. 3.1.1 [27]. We used the predictors that are considered physiologically important for plants [28] and that were not strongly correlated with each other (Pearson correlation coefficient, r^2^ <|0.80|). To model species distributions, we used the following six variables: BIO3, isothermality; BIO4, temperature seasonality; BIO14, precipitation of driest month; BIO19, precipitation of coldest quarter; the presence/absence of suitable substrate. We removed duplicates using ENMTools [29]. Finally, 360 for *S*. *cordifolia* and 223 presence records for *V*. *argenteria* were applied in SDM analysis.

To account for model-based uncertainties, we applied six SDM techniques: multivariate adaptive regression splines (MARS–[30]), generalized linear models (GLM–[31]), classification tree analysis (CTA–[32]), flexible discriminant analysis (FDA–[33]), random forest (RF–[34]), and Maximum entropy modelling (MaxEnt–[35]). These techniques belong to three different categories of models (i.e. regression methods–MARS and GLM; classification methods–CTA and FDA; and machine learning algorithms–RF and MaxEnt).

Analyses were implemented with BIOMOD2 v. 1.3.5 package [36] for R v. 3.1.1 [27]. The number of replicate sets of pseudo-absences may influence the predictive accuracy of the models. For this reason, we chose the best strategy in pseudo-absences selection according to Barbet-Massin *et al*. [37] tips (S2 Table). The predictive performance of the models was evaluated for each pseudo-absence run using a random subset (70%) of the initial dataset each time to calibrate the models, while the remaining 30% were used to evaluate the models. The predictive performance of the models was evaluated based on the true skill statistic (TSS), which takes into account both omission and commission errors and it is not affected by prevalence [38]. To transform the inferred continuous probability values to binary presence-absence form we used the TSS. Consensus projection under current conditions was created summing binary predictions of the six modelling techniques. We also created consensus projection under LGM conditions summing the binary predictions of the six modelling techniques for the three LGM climates, thus outlining those areas predicted by most modelling techniques under the different climates.

### DNA sampling and sequencing

A total of 94 individuals from 19 populations for *S*. *cordifolia* and a total of 80 individuals from 21 populations for *V*. *argenteria* were sampled, covering the entire geographical range of species (Table 1). Permissions to collect samples in field were obtained from Parc National du Mercantour and form Parco Naturale Alpi Marittime. Total DNA was extracted from leaf tissue using the DNeasy Plant Mini Kit (Qiagen AG, Hombrechtikon, Switzerland), following the manufacturer’s instructions. Seven cpDNA regions in *S*. *cordifolia* (*rps16* gene, *trnQ-rps16*, *trnG2G-trnG*, *trnH-psbA*, *trnC-ycf6R*, *trnT*_*a*_*-trnL*_*b*_, *trnL*-*trnF*) and seven cpDNA regions in *V*. *argenteria* (*atpF-atpH*, *rpoC1* gene, *rps12-rpL20*, *trnG2G-trnG*, *trnH-psbA*, *trnL-trnF*, *trnT*_*a*_*-trnL*_*b*_) were chosen (S3 Table). PCR reactions were performed using Eppendorf thermocycler, with 20 μL total volume reactions containing 5–30 ng DNA, 10 μL GoTaq Green Master Mix (Promega, Milan, Italy), 10 pmol of each primer and purified water. We used the following PCR conditions: 2 min pre-treatment at 94°C, followed by 35 cycles of 120 s denaturation (94°C), 45 s annealing (48–63°C), 75–120 s extension (72°C). After the last cycle the temperature was kept at 72°C for the last 7 min of extension and then lowered to 4°C (S3 Table). The PCR products were checked on 2% agarose gels in biotium staining. The amplification products were then purified using the commercial kit QIAquick PCR Purification Kit (Qiagen, USA). All DNA sequencing was performed by Macrogen company (Korea). Sequences were aligned using MAFFT v. 6.903 [39] and adjusted by hand. The cpDNA regions were concatenated in a single matrix. We considered indels as character because previous studies exploring the utility of cpDNA indels have concluded that they are informative and that they might increase resolution between geographical regions of high diversity [40]. All sequences have been deposited in GenBank database (S4 Table).

**Table 1:** Information about the populations of *S*. *cordifolia* and *V*. *argenteria* sampled for the analysis. Population code (C), Latitude N (Lat), Longitude E (Long) and elevation (Elev.) as metres above sea level, are reported. Populations from Corsica were sampled in Florence Herbarium (FI) and the coordinates are approximate.

Taxon	Population	C	Lat	Long	Elev.
*S*. *cordifolia*	Vallon de Rabuons, Vallèe du Tinée, FR	1	44.2704	6.96283	2400
	L'illions, Vallée du Var, FR	2	44.0490	6.95815	1638
	Vallone di Sant'Anna, Valle Stura, IT	3	44.2735	7.13497	1341
	S. Anna di Vinadio, Valle Stura, IT	4	44.2264	7.10274	2154
	Isola 2000, Vallèe du Tinée, FR	5	44.2069	7.11142	1715
	La Barre-Roure, Vallèe du Tinèe, FR	6	44.1015	7.08885	1378
	Mt. Pépoiri, Vallèe du Tinèe, FR	7	44.1141	7.18859	2315
	Vallone del Valasco, Val Gesso, IT	8	44.2143	7.23606	2440
	Vallone della Meris, Val Gesso, IT	9	44.2439	7.22745	2300
	L. di Fremamorta, Val Gesso, IT	10	44.1745	7.25362	2107
	M. Ray, Val Gesso, IT	11	44.2262	7.36828	1971
	Bacino del Chiotas, Val Gesso, IT	12	44.1700	7.33278	2095
	Rifugio Soria-Ellena, Val Gesso, IT	13	44.1401	7.36359	1884
	Col de Fenestre, Vallée du Vésubie, FR	14	44.1082	7.35565	2314
	V. de la Madone de Fenestre, V. Vésubie, FR	15	44.0841	7.28705	1314
	St. Grat de Gordolasque, V. Vésubie, FR	16	44.0727	7.39797	1775
	Lago Veil del Buc, Val Gesso, IT	17	44.1491	7.42930	2444
	Mt. Peirafica, Vallée de la Roya, FR	18	44.1297	7.51889	1990
	Ref. Merveilles, Vallée de la Roya, FR	19	44.0563	7.46058	2097
*V*. *argenteria*	Ref. Lausa, Vallèe du Tinèe, FR	1	44.2978	6.96232	2544
	Vallon de Rabuons, Vallèe du Tinèe, FR	2	44.2607	6.98320	2678
	Collalunga, Valle Stura, IT	3	44.2235	7.03190	2445
	Tesina/Sabulé, Valle Stura, IT	4	44.2271	7.08179	2466
	Mt Saint Sauveur, Vallèe du Tinèe, FR	5	44.1700	7.13032	2414
	Bassa del Druos,	6	44.1902	7.19138	2610
	Col de Barn-Mt. Pepoiri, Vallèe du Tinèe, FR	7	44.1140	7.20159	2493
	Alta Valle Meris, Val Gesso, IT	8	44.2319	7.21665	2620
	Lago inferiore di Valrossa, Val Gesso, IT	9	44.2161	7.22214	2507
	Laghi Colle di Fremamorta, Val Gesso, IT	10	44.1601	7.25121	2433
	Altopiano del Baus, Val Gesso, IT	11	44.1741	7.31499	2644
	Valle del Brocan, Val Gesso, IT	12	44.1526	7.31649	2504
	Passo delle Finestrelle, Val Gesso, IT	13	44.1572	7.34966	2433
	Col de Fenestre, Vallée du Vésubie, FR	14	44.1130	7.36051	2384
	C. Vallette de Prals, Vallée du Vésubie, FR	15	44.0639	7.35972	2385
	Rifugio Pagari, ValGesso, IT	16	44.1226	7.40698	2597
	Monte Carboné, Val Gesso, IT	17	44.1520	7.43214	2654
	P. dell'Arpette, Vallée de la Roya, FR	18	44.0599	7.42640	2528
	Monte Rotondo, Corse, FR*	19	42.2200	9.07000	2400
	Monte d'Oro, Corse, FR**	20	42.1400	9.10000	2100
	Monte Renoso, Corse, FR***	21	42.0600	9.13000	2500

* Monte Rotondo, 7.1907, Martelli (FI)

** Monte d’Oro, 25.7.1907, Martelli (FI)

*** Monte Renoso, 2500 m, Levriere (FI).

### Genetic diversity and differentiation

Haplotype number (H), haplotype diversity (H_d_), number of segregating sites (S) and nucleotide diversity (π) [41] were calculated with DNASP 4.10 [42]. The total gene diversity (H_T_), population differentiation (G_ST_) and an estimation of population subdivision for phylogenetically ordered alleles (N_ST_) were calculated using the program PERMUT [43]. We further tested the presence of phylogeographical structure (*sensu* [43]), comparing N_ST_ to G_ST_ using 1000 permutations to obtain statistical significance with the program PERMUT. To evaluate the genetic structure, we conducted an analysis of molecular variance (AMOVA) in ARLEQUIN 3.1 [44], partitioning the genetic diversity into the diversity among groups, the diversity among populations within groups, and the diversity within populations. The correlation between the genetic (F_ST_) and geographic pairwise population distance matrices was evaluated using a Mantel test [45] with 10,000 permutations using ARLEQUIN 3.1. The Mantel test was calculated on all populations analysed both in *S*. *cordifolia* and *V*. *argenteria* and on the Maritime and Ligurian Alps’ populations only in *V*. *argenteria*.

### Analysis of haplotypes

To determine the genetic relationship among populations, we followed the method implemented by Muñoz-Pajares [46] in the SIDIER package. The method gathers the evolutionary information contained in insertions and deletions (indels) and combines such information with the p-distance matrix inferred from substitutions. This method improves the resolution of phylogenetic inference of low-divergence organisms, yielding results compatible with other evolutionary procedures [46]. Based on recommendations by Simmons *et al*. [47], indels in the concatenated dataset were coded as single mutational event using a variation of modified complex indel coding (MCIC—*sensu* [48]).

Spatial analysis of molecular variance (SAMOVA—[49], http://web.unife.it/progetti/genetica/Isabelle/samova.html) was used to identify groups of populations that are geographically homogeneous and maximally differentiated from each other without assuming any prior association between populations. The algorithm identifies the optimal number of groups of populations (k) by maximizing F_CT_ (the proportion of total genetic variance due to differences among groups of populations) and minimizing F_SC_ (genetic differentiation among populations within groups). SAMOVA was run for 10,000 iterations from each of 100 random initial conditions, and testing the predefined number of groups (k) from 2 to 10.

Best-fit models of nucleotide substitution were determined to be a TIM1 + I model for all *S*. *cordifolia* sequences and F81 model for *V*. *argenteria*, by the Akaike Information Criterion using JMODELTEST 2.1.1 [50]. Divergence time among haplotypes was estimated using BEAST v.1.8.0 [51]. We used a strict molecular clock and a coalescent Gaussian Markov random field (GMRF) Bayesian skyride tree prior. We sampled all parameters once every 2000 steps from 1 x 10^9^ MCMC steps after a burn-in of 2.5 x 10^8^ steps. The program TRACER [52] was used to examine convergence of chains to the stationary distribution [effective sample size (ESS) > 200]. In this study, we used normal distribution priors with a mean of 2 x 10^−9^ and a standard deviation of 5 x 10^−10^ substitutions per site per year to cover the cpDNA substitution rates for angiosperm (1–3 x 10^−9^ substitutions per site per year; [53, 54, 55]).

## Results

### Species distribution models

**Fig 2:**
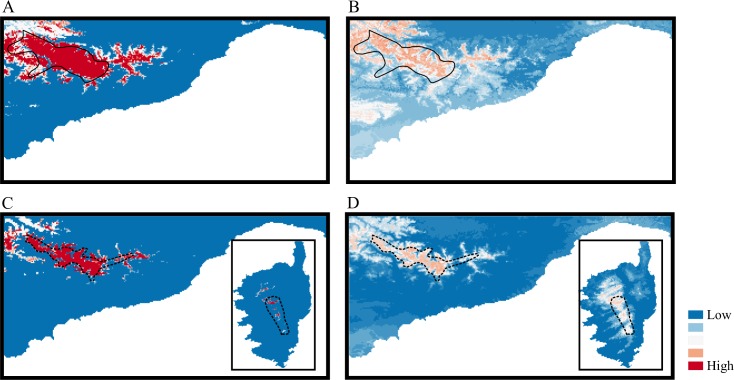
Maps showing the consensus among the 6 binary maps of the current distribution (A and C) and of the 18 binary maps of the Last Glacial Maximum distribution (B and D; LGM ~ 21 kya) for *S*. *cordifolia* (A and B) and *V*. *argenteria* (C and D). Models were obtained using six algorithms (MARS, GLM, CTA, FDA, RF and MAX). LGM models are obtained using three palaeoclimate data (MIROC, CCSM and MPI). The inferred continuous probability values were converted to binary using TSS threshold. Red indicates greatest consensus among models and blue indicates lowest consensus among models. Current known distribution area of *S*. *cordifolia* (continuous black line) and *V*. *argenteria* (dotted black line) is reported.

Under current climate conditions, following Swets’ scale modified by Araújo *et al*. [56], TSS (Table 2) indicated excellent model performance for both species. The SDMs projections for current conditions (Fig 2A and 2C) roughly fitted the current species distribution. The predicted potential distribution during the LGM was different among climates and algorithms (S1 Fig). In both species, LGM models based on CCSM and MIROC climate identified regions similar to present models, but reduced in range. On the contrary, MPI climate predicted a range wider than present in *S*. *cordifolia* while in *V*. *argenteria* it predicted a range wider than present in Corsica and narrower in the Maritime and Ligurian Alps. Despite this variation, the ensemble maps of all climates and algorithms suggested the presence of suitable areas in the current distributional range (Fig 2B and 2D).

**Table 2:** Evaluation of individual modelling techniques for *S*. *cordifolia* and *V*. *argenteria*. Statistics given are the mean values and the associated standard deviations (in brackets) for the true skill statistic (TSS). Values given are. Accuracy classification for TSS following Swets’ scale modified by Araújo *et al*. [56]: 1> excellent>0.8>good>0.6>fair>0.4>poor>0.2>fail. MARS Multiple Additive Regression Spline; GLM, Generalized Linear Models; CTA, Classification Tree Analysis; FDA, Flexible Discriminant Analysis; RF, Random Forest; MAX, Maximum Entropy.

	*S*. *cordifolia*	*V*. *argenteria*
	TSS	TSS
MARS	0.887 (0.053)	0.932 (0.077)
GLM	0.893 (0.008)	0.931 (0.021)
CTA	0.938 (0.014)	0.940 (0.033)
FDA	0.929 (0.050)	0.963 (0.033)
RF	0.975 (0.013)	0.972 (0.019)
MaxEnt	0.887 (0.013)	0.941 (0.016)

### Sequence data

The total combined length of the aligned sequences of the seven cpDNA was 4461 bp in *S*. *cordifolia* and 3730 bp in *V*. *argenteria*. We detected variations in four out of seven cpDNA regions both in *S*. *cordifolia* and in *V*. *argenteria* (Table 3). In particular, we recorded 12 variable and 9 parsimony-informative sites in *S*. *cordifolia* and 15 variable and parsimony-informative sites in *V*. *argenteria*. Alignments of the sequences of *S*. *cordifolia* and *V*. *argenteria* showed 1 and 17 insertion/deletion sites (indels) respectively. In all, there were 13 variable sites among the sampled individuals of *S*. *cordifolia* and 65 in *V*. *argenteria* (S5 Table). We recovered 12 haplotypes within *S*. *cordifolia* and 16 haplotypes within *V*. *argenteria* (Table 3).

**Table 3:** Chloroplast genetic diversity within *S*. *cordifolia* and within *V*. *argenteria*. Number of sampled individuals (N_I_), number of haplotypes (h), number of private haplotypes (ph), haplotype diversity (H_d_) and nucleotide diversity (π) are indicated for populations from each species.

Taxon/population	N_I_	h	ph	H_d_	π
*S*. *cordifolia*					
1	5	1	0	0	0
2	5	2	1	0.4	0.00027
3	5	1	0	0	0
4	5	2	1	0.4	0.00018
5	5	2	0	0.7	0.00031
6	5	3	0	0.7	0.00027
7	5	1	0	0	0
8	5	1	0	0	0
9	4	4	1	1	0.00086
10	4	4	1	1	0.00101
11	5	2	0	0.6	0.00013
12	6	2	0	0.6	0.00013
13	5	2	0	0.6	0.00013
14	5	2	0	0.6	0.00081
15	5	1	0	0	0
16	5	2	1	0.4	0.00018
17	5	1	0	0	0
18	5	1	0	0	0
19	5	1	0	0	0
	94	12		0.824	0.00089
*V*. *argenteria*					
1	4	1	0	0	0
2	4	1	0	0	0
3	5	1	0	0	0
4	5	2	1	0.6	0.00049
5	6	1	0	0	0
6	5	1	0	0	0
7	5	3	2	0.7	0
8	3	1	0	0	0
9	2	1	0	0	0
10	3	1	0	0	0
11	5	2	1	0.6	0.00016
12	4	4	2	1	0.00036
13	5	2	0	0.4	0.00022
14	3	1	0	0	0
15	5	1	0	0	0
16	3	2	1	0.7	0
17	4	2	1	0.5	0
18	3	1	1	0	0
19	2	1	1	0	0
20	2	1	1	0	0
21	2	1	1	0	0
	80	16		0.834	0.00081

### Phylogeographical relationships and divergence times of haplotypes

**Fig 3:**
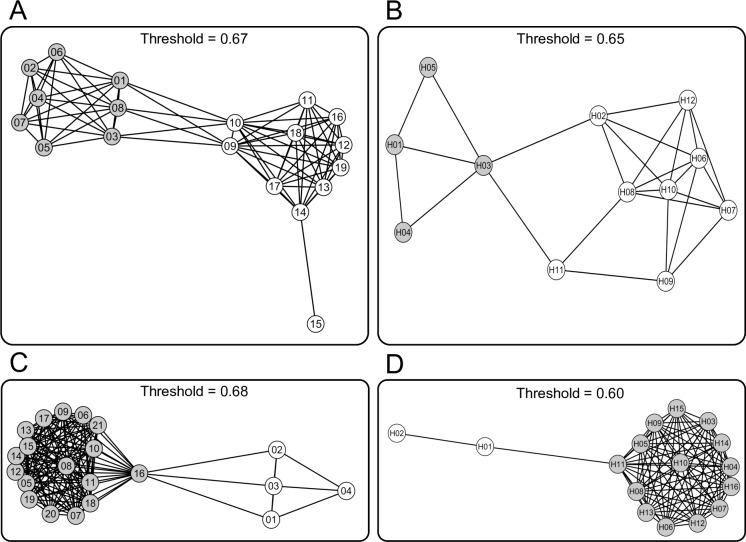
Percolation network obtained combining distance matrices of indels and substitutions. Relationships among populations (A and B) and haplotypes (C and D) are showed for *S*. *cordifolia* (A and C) and *V*. *argenteria* (B and D). The network was built connecting distances lower than the estimated percolation threshold (reported in the figure). Groups (i.e., subsets of nodes conforming densely connected subgraphs) are represented in different grey tones.

The dendrogram shows a clear divergence between two groups of haplotypes in both species. In *S*. *cordifolia* the first group (grey haplotypes in Fig 3A) is present in the north-western populations while the second group (white haplotypes in Fig 3C) is present throughout the entire range of distribution. In *V*. *argenteria* the first group (grey haplotypes in Fig 3B) is present in the north-western populations while the second group (white haplotypes in Fig 3D) is only present in the south-eastern populations.

**Fig 4:**
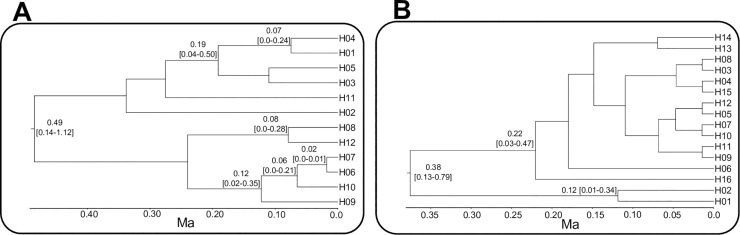
BEAST derived chronogram for haplotypes in *S*. *cordifolia* (A) and *V*. *argenteria* (B) based on cpDNA regions. Mean divergence time and 95% Highest Posterior Density (HPD) intervals are reported for nodes with posterior probability higher than 50%. Haplotype codes correspond to those in Table 1.

Diversification of *S*. *cordifolia* and *V*. *argenteria* haplotype lineages occurred during the Pleistocene, with the main lineages diverging during the middle Pleistocene. Phylogenetic analysis of cpDNA haplotypes showed that *S*. *cordifolia* differentiated into two main lineages at 0.49 (0.14–1.12) Ma. The first (H01-H05, H11) is mainly present in south-eastern populations (H01-H04) but also in some north-western populations (H05 and H11) while the second lineage (H06-H10, H12) is mainly present in north-western populations (H06-H07, H09-H10, H12) but also in some south-eastern populations (H08). Similarly, *V*. *argenteria* differentiated into two main lineages at 0.37 (0.12–0.79) Ma. The first lineage (H01 and H02) is exclusive of the north-western populations while the second lineage (H03-H16) is exclusive of the south-eastern and Corsican populations. However, the node age estimates should be interpreted with caution given the width of the 95% confidence intervals for each node (Fig 4).

### Genetic diversity and differentiation of populations

In *S*. *cordifolia* eight populations contained just one haplotype, seven populations contained two haplotypes, two populations contained three haplotypes and the remaining two populations contained four haplotypes. Five populations distributed throughout the range of the species showed a private haplotype (2, 4, 9, 10 and 16). The populations showing the higher values of haplotype diversity were located in the central-northern part of distribution range. The total gene diversity was high (H_T_ = 0.847, SE = 0.0251) while the within-population diversity was low (H_S_ = 0.368, SE = 0.0824). The differentiation among populations based on cpDNA variation (G_ST_ = 0.565, SE = 0.0960) indicates a moderate degree of population genetic structure within *S*. *cordifolia*. Allelic differentiation (N_ST_ = 0.749, SE = 0.0835) is greater than but not significantly different from G_ST_, indicating a weak genetic differentiation among populations. In *V*. *argenteria* 14 populations contained just one haplotype, five populations contained two haplotypes and the remnant two populations contained three and four haplotypes, respectively. Ten populations, mainly located in the southern part of Maritime Alps and in Corsica, showed private haplotype (4, 7, 11, 12, 16, 17, 18, 19, 20 and 21). The populations showing the higher values of haplotype diversity were located in the central-southern part of distribution range. The total gene diversity was high (H_T_ = 0.885, SE = 0.0417) while the within-population diversity was low (H_S_ = 0.213, SE = 0.0710). The differentiation among populations based on cpDNA variation (G_ST_ = 0.760 SE = 0.0785) indicates a large degree of population genetic structure within *V*. *argenteria*. Allelic differentiation (N_ST_ = 0.921 SE = 0.0486) is greater than and significantly different from G_ST_, indicating a strong genetic differentiation among populations. AMOVA (Table 4) showed that a large proportion (*S*. *cordifolia*: 77.32%; *V*. *argenteria*: 87.70%) of the chloroplast variation was partitioned among the population groups identified by SAMOVA. In contrast, the component of among-populations variation within groups was the lowest (*S*. *cordifolia*: 7.09%; *V*. *argenteria*: 4.74%). The proportion of variation residing within populations (*S*. *cordifolia*: 15.59%; *V*. *argenteria*: 7.56%) was consistent with the relatively low value of within-population diversity. Mantel test revealed a significant isolation-by-distance pattern in both species (*S*. *cordifolia*: r = 0.417, P < 0.0001; *V*. *argenteria* all populations: r = 0.296, P < 0.0001; *V*. *argenteria* Maritime and Ligurian Alps populations: r = 0.586, P < 0.0001).

**Table 4:** Results of AMOVA of cpDNA data from 19 populations of *S*. *cordifolia* and 21 populations of *V*. *argenteria*. df: degree of freedom; SS: sum of square; variation %: percentage of total variance; ** P<0.001.

Species	Source of variation	df	SS	Variation%	F statistic
*S*. *cordifolia*					
	Among population	18	170.409	77.96**	F_ST_ = 0.77957
	Within population	75	38.400	22.04	
	Among SIDIER groups	1	117.707	70.65**	F_CT_ = 0.70655
	Among population within groups	17	52.702	14.84**	F_SC_ = 0.50558
	Within population	75	38.400	14.51**	F_ST_ = 0.85491
	Among SAMOVA groups	2	143.813	77.32**	F_CT_ = 0.77320
	Among population within groups	16	26.595	7.09**	F_SC_ = 0.31259
	Within population	75	38.400	15.59**	F_ST_ = 0.84409
*V*. *argenteria*					
	Among population	20	768.596	90.20**	F_ST_ = 0.90196
	Within population	59	63.217	9.80	
	Among SIDIER groups	1	399.431	69.37**	F_CT_ = 0.69369
	Among population within groups	19	369.165	25.12**	F_SC_ = 0.82023
	Within population	59	63.217	5.51**	F_ST_ = 0.94493
	Among SAMOVA groups		707.960	85.18**	F_CT_ = 0.85175
	Among population within groups		60.636	7.69**	F_SC_ = 0.51905
	Within population		63.217	7.13**	F_ST_ = 0.92870

**Fig 5:**
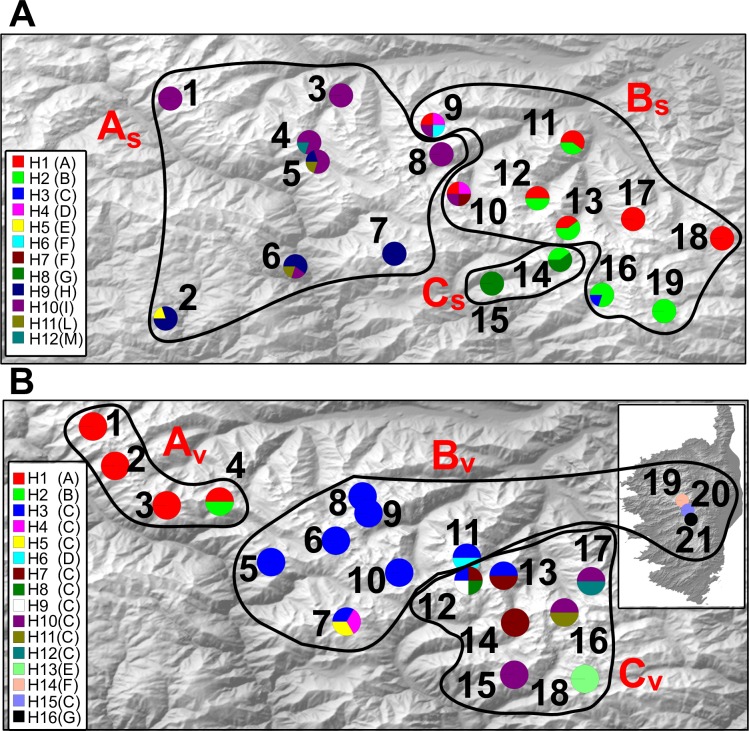
Geographical distribution of haplotypes across sampled populations of *S*. *cordifolia* (A) and *V*. *argenteria* (B). Pie charts represent haplotype proportions. Haplotype codes correspond to those in Table 1. Population groups identified by SAMOVA are delimited by continuous black lines and named by capital red letters for *S*. *cordifolia* (A_S_-C_S_) and *V*. *argenteria* (A_V_-D_V_).

SAMOVA analysis detected 3 groups in *S*. *cordifolia* (A_S_, B_S_ and C_S_ in Fig 5) and *V*. *argenteria* (A_V_, B_V_ and C_V_ in Fig 5). Although the F_CT_ continued to increase slightly and the F_SC_ continued to decrease slightly as the number of groups was increased for each species, the F_CT_ largely plateaued and the F_SC_ markedly decreased at these respective groupings (S2 Fig). Basing on population distance, SIDIER analysis clearly separates populations into two discrete clusters: north-western and south-eastern populations (Fig 3A and 3B) in both species. In particular, in *S*. *cordifolia* the north-western cluster corresponds to SAMOVA group A_S_ while south-eastern cluster corresponds to B_S_ and C_S_ groups. Similarly, in *V*. *argenteria* the north-western cluster corresponds to SAMOVA group A_V_ while south-eastern cluster corresponds to B_V_ and C_V_ groups of SAMOVA.

## Discussion

### Palaeoclimatic models and haplotype divergence

In general, Alpine species are thought to have persisted LGM (0.12–0.01 Ma–[16, 57]) in glacial refugia and later to have recolonised newly available areas [4, 6]. As a consequence, the LGM had a strong influence on the genetic structure, distribution and evolution of plant species [2, 6, 58]. In fact, the LGM is recognized as a primary factor causing distributional patterns of endemic plants in Alps [5] while, the current ranges of many Alpine plants may also have been shaped by delayed Holocene recolonisation of suitable sites from refugial areas [59].

The palaeo-distribution models inferred in this work together with the low and patchy ice sheet coverage of LGM in the Maritime and Ligurian Alps [60, 61] suggest that the two study species, *S*. *cordifolia* and *V*. *argenteria*, probably survived the LGM in the sites where they were already present (*in situ* survival *sensu* [57]). The weak impact of the LGM in Maritime and Ligurian Alps may have been due to the climatic mitigation effect of the nearby Mediterranean Sea and to the relatively steep relief of this sector which was probably unfavourable to the formation of major glacier basins [61]. Recently, Patsiou *et al*. [62] showed that *Saxifraga florulenta* Moretti, endemic to Maritime Alps, survived the last glaciation in several microrefugia close to sites of current occurrence. Our finding supports the idea of a weak impact of the LGM on the distribution pattern of endemic species in the Maritime and Ligurian Alps, in marked contrast to its probable effect in other parts of the Alps [2, 4]. In line with the possibility of *in situ* survival during the LGM detected by SDM and previously debated, our analyses (Fig 4) suggest a divergence time between lineages predating the Late Pleistocene glaciation (0.115–0.018 Ma) both in *S*. *cordifolia* (0.490 Ma; 95% HPD:0.140–1.120 Ma) and *V*. *argenteria* (0.370 Ma; 95% HPD: 0.120–0.790 Ma). We thus suggest that these two species illustrate the persistence of an ancient genetic structure through several Pleistocene climatic cycles. A similar time frame has been previously suggested for other animal and plant species from other biogeographical regions [63, 64].

Our results are also congruent with the palaeo-climatic reconstructions. In fact, the most extensive glaciation in Mediterranean mountains occurred during the Middle Pleistocene (0.780–0.120 Ma), in particular a major phase probably occurred during Mindel glaciation (0.470–0.420 Ma–[65, 66]). Moreover, pollen-inferred historical climate indicates that maxima temperatures for interglacial stages in the Middle Pleistocene (0.780–0.120 Ma) were similar to the lowest values observed during the Late Pleistocene glacial period (0.115–0.018 Ma–[67]). In contrast, in the central Alps there is evidence of extensive glaciation during Riss (0.190–0.115 Ma–[68]) and Würm glaciation (0.115–0.018 Ma–[60]). Although the differentiation between main lineages is most likely to have begun in the Middle Pleistocene, haplotype divergence within main lineages probably occurred successively (Fig 4). This is congruent with the observation that in some Alpine animal species the separation of the major clades occurred long before the LGM and the following repeated glacial and interglacial periods may have been the motor for marked differentiation that is currently observed [69, 70]. A similar time frame was detected in another endemic species in the calcareous cliffs of the Maritime and Ligurian Alps, *P*. *allionii* where the separation into two groups dates back to the Middle Pleistocene [13]. In contrast, in *S*. *florulenta* the differentiation of two ancestral gene pools seems to have been influenced by range contractions that were more recent than the glacial oscillations [20], an interpretation that is supported by SDMs [62]. Nevertheless, the difference in differentiation time for *S*. *florulenta* compared to those of our study species agrees with the observation that, although recurrent patterns exist, some species may exhibit distinct patterns reflecting the unique, rather than the shared, aspects of species’ histories [71, 72].

In general, our results for *S*. *cordifolia* and *V*. *argenteria* point out the importance of not just the LGM but also previous glacial cycles in shaping the contemporary genetic structure of plant species in the Maritime and Ligurian Alps. The results thus corroborate the notes inferring the necessity to go beyond interpretations that relate only to the role of a small number of major historical events, such as the LGM and the Messinian Salinity Crisis [7].

### The distinctiveness of current populations

In general, current patterns of genetic variation are explained by contraction/expansion model in association with climatic oscillations. In this model refuge populations are associated with multiple genetic lineages and higher genetic diversity compared to more recently established populations [3]. Due to the recurrent founder effect affecting colonizing populations, the genetic diversity is supposed to decrease by increasing the distance from refugia, producing a genetic signature [73]. Nevertheless, this pattern can be obscured by secondary contact of vicariant lineages which results in admixing populations with high heterozygosity and genetic diversity [74].

The total gene diversity and haplotype diversity (Table 2) detected in *S*. *cordifolia* (H_T_ = 0.847; H_d_ = 0.824) and *V*. *argenteria* (H_T_ = 0.885; H_d_ = 0.834) are similar and the total gene diversity is higher than the mean value (H_T_ = 0.670; [64]) detected in 170 of the plant species studied by Petit *et al*. [74]. Comparably, high total gene diversity and haplotype diversity have been detected in several other endemic species (e.g., *Phyllodoce nipponica* Makino: H_T_ = 0.852, [75]; *Rhodiola dumulosa* (Franchet) S.H. Fu: H_T_ = 0.981, [76]; 2010; *Arenaria provincialis* Chater & Halliday: H_d_ = 0.94, [77]). Although both species show relatively high genetic differentiation among populations, the within-population values of haplotype and nucleotide diversity are lower and the number of private haplotypes higher in *V*. *argenteria* than in *S*. *cordifolia* (Table 3), resulting in a higher level of genetic differentiation between populations and in a more marked phylogeographical structure (*S*. *cordifolia*: N_ST_ = 0.749; G_ST_ = 0.565; *V*. *argenteria*: N_ST_ = 0.921; G_ST_ = 0.760, see also Table 4). Low within population diversity we observed suggests bottleneck or founder effects during glacial population contraction and post-glacial expansions, resulting in few haplotype being fixed in most populations. This result, together with the weak phylogeographical structure, also suggests that a repeated pattern of contraction/expansion, throughout the glacial cycles, may have shaped the contemporary genetic structure of the studied species. This result is in line with our SDM and molecular dating findings. The level of genetic differentiation detected among populations of both species even indicates low levels of recurrent gene flow among populations, as also confirmed by IBD results. The high haplotype and gene diversity, taken together with the patchy distribution and the numerous private haplotype, may be explained by a long history of differentiation and *in situ* glacial survival. Two factors may contribute to the higher cpDNA-based population subdivision in *V*. *argenteria* compared to *S*. *cordifolia*. First, vicariance events due to glaciation cycles may have had a stronger impact on *V*. *argenteria*, growing at higher altitude. Second, a more efficient seed dispersal mechanism in *S*. *cordifolia* may contribute to the lack of correspondence between population groups and haplotype clades in this species. This last explanation seems unlikely because *V*. *argenteria* may be dispersed by bird (see discussion below). However, the IBD result suggests a similar immigration by seed in Maritime and Ligurian Alps populations of both species (*S*. *cordifolia*: r = 0.417, P < 0.0001; *V*. *argenteria*: r = 0.586, P < 0.0001). In addition, the high proportion of variation residing among the population groups identified by SAMOVA indicates that past climatic oscillations would have stimulated a stronger intraspecific diversification and population genetic differentiation in *V*. *argenteria*. According to this result, the higher within-population diversity detected in *S*. *cordifolia* may be due to its larger altitudinal range. This may have facilitated short-distance altitudinal migrations likely decreasing the impact of founder effects and the genetic signature of glaciations. A similar pattern of short-distance altitudinal shift and recurrent re-colonizations of neighbouring populations was reported in *Armeria* genus growing at high altitude [78]. This is congruent with the higher haplotype diversity observed in central populations for *S*. *cordifolia* and in southern populations for *V*. *argenteria*, and with the stronger effect of glaciation on the latest. *V*. *argenteria* growing only in the alpine belt probably survived glaciation in a southern peripheral refugium while *S*. *cordifolia* may have survived to last glaciation in several refugia at lower altitudes, showing so its higher diversity in the contact zone.

The recent origin of haplotypes detected in Corsica suggests that the disjoint Corsican populations of *V*. *argenteria* may have originated by a rare long-distance dispersal of seed by birds rather than by vicariance due to Corsica-Sardinia microplate disjunction (15 Ma). In fact, seeds of violets have been shown to be capable of bird-mediated endozoochorous dispersal [79]. Moreover, Corsica is located along one of the main present-day migration routes of birds from European continental regions to the African areas [80]. This random dispersion is congruent with the weak IBD detected among all populations of *V*. *argenteria* (r = 0.296, P < 0.0001).

The complex phylogeographical pattern observed in the studied species would indicates that, even if some species-specific differences are detected, both population and genotype extinction was minimal during the LGM, probably due to the low impact of glaciations and to topographic complexity in this area [81]. This result is in line with evidences from SDM and divergence time among haplotypes. In the south western Alps, survival in peripheral refuges and nunataks during the LGM has recently been demonstrated in *Primula* spp. using both SDM and phylogeographical approaches [12]; however, our results are congruent with previous findings in the Maritime and Ligurian Alps where glaciations seem to have had a low influence on plant distribution and their effect seems to be weakened by high level of postglacial migrations [81]. These findings are more similar to that expected for a Mediterranean mountain region [7, 8] rather than for an Alpine region [16, 58], where patterns were simplified to a great extent by major losses of diversity during glacial periods.

## Supporting Information

S1 Table Pearson correlation coefficient between the 19 bioclimatic predictors. (DOCX)

S2 Table The number of replicate sets of pseudo-absences. (DOCX)

S3 Table cpDNA regions in *S*. *cordifolia* PCR. (DOCX)

S4 Table Sequences deposited in GenBank database. (DOCX)

S5 Table Variable sites among the sampled individuals of *S*. *cordifolia* and *V*. *argenteria*. (DOCX)

S1 Fig LGM distributions models. (DOCX)

S2 Fig Results of the spatial analyses of molecular variance (SAMOVA). (DOCX)
