# Supplementary material for: Phylogeographic Insights into a Peripheral Refugium: The Importance of Cumulative Effect of Glaciation on the Genetic Structure of Two Endemic Plants
Source: PLoS One. 2016 Nov 21;11(11):e0166983. doi: 10.1371/journal.pone.0166983 (PMC5117763; doi:10.1371/journal.pone.0166983)
Supplement: S1 Table — (DOCX) [file pone.0166983.s001.docx]

**S1 TABLE.** Pearson correlation coefficient between the 19 bioclimatic predictors.

|  | bio1 | bio2 | bio3 | bio4 | bio5 | bio6 | bio7 | bio8 | bio9 | bio10 | bio11 | bio12 | bio13 | bio14 | bio15 | bio16 | bio17 | bio18 | bio19 |
| --- | --- | --- | --- | --- | --- | --- | --- | --- | --- | --- | --- | --- | --- | --- | --- | --- | --- | --- | --- |
| bio1 | 1.000 |  |  |  |  |  |  |  |  |  |  |  |  |  |  |  |  |  |  |
| bio2 | 0.332 | 1.000 |  |  |  |  |  |  |  |  |  |  |  |  |  |  |  |  |  |
| bio3 | 0.531 | 0.878 | 1.000 |  |  |  |  |  |  |  |  |  |  |  |  |  |  |  |  |
| bio4 | -0.373 | 0.298 | -0.175 | 1.000 |  |  |  |  |  |  |  |  |  |  |  |  |  |  |  |
| bio5 | 0.976 | 0.492 | 0.603 | -0.185 | 1.000 |  |  |  |  |  |  |  |  |  |  |  |  |  |  |
| bio6 | 0.960 | 0.088 | 0.380 | -0.575 | 0.877 | 1.000 |  |  |  |  |  |  |  |  |  |  |  |  |  |
| bio7 | -0.038 | 0.793 | 0.413 | 0.811 | 0.179 | -0.315 | 1.000 |  |  |  |  |  |  |  |  |  |  |  |  |
| bio8 | 0.923 | 0.313 | 0.412 | -0.146 | 0.930 | 0.853 | 0.090 | 1.000 |  |  |  |  |  |  |  |  |  |  |  |
| bio9 | 0.887 | 0.271 | 0.448 | -0.328 | 0.860 | 0.853 | -0.046 | 0.802 | 1.000 |  |  |  |  |  |  |  |  |  |  |
| bio10 | 0.995 | 0.358 | 0.516 | -0.287 | 0.987 | 0.937 | 0.032 | 0.939 | 0.883 | 1.000 |  |  |  |  |  |  |  |  |  |
| bio11 | 0.987 | 0.241 | 0.505 | -0.514 | 0.931 | 0.987 | -0.182 | 0.879 | 0.875 | 0.969 | 1.000 |  |  |  |  |  |  |  |  |
| bio12 | -0.874 | -0.056 | -0.278 | 0.431 | -0.815 | -0.908 | 0.249 | -0.827 | -0.743 | -0.865 | -0.886 | 1.000 |  |  |  |  |  |  |  |
| bio13 | -0.447 | 0.046 | -0.127 | 0.307 | -0.403 | -0.509 | 0.245 | -0.451 | -0.320 | -0.436 | -0.471 | 0.775 | 1.000 |  |  |  |  |  |  |
| bio14 | -0.900 | -0.004 | -0.282 | 0.585 | -0.809 | -0.960 | 0.368 | -0.783 | -0.799 | -0.877 | -0.937 | 0.928 | 0.546 | 1.000 |  |  |  |  |  |
| bio15 | 0.832 | -0.126 | 0.213 | -0.722 | 0.705 | 0.935 | -0.522 | 0.679 | 0.737 | 0.792 | 0.894 | -0.812 | -0.400 | -0.937 | 1.000 |  |  |  |  |
| bio16 | -0.601 | -0.093 | -0.191 | 0.136 | -0.594 | -0.601 | 0.056 | -0.658 | -0.476 | -0.612 | -0.585 | 0.850 | 0.939 | 0.616 | -0.418 | 1.000 |  |  |  |
| bio17 | -0.888 | 0.053 | -0.239 | 0.612 | -0.787 | -0.963 | 0.418 | -0.767 | -0.781 | -0.862 | -0.930 | 0.928 | 0.556 | 0.995 | -0.958 | 0.611 | 1.000 |  |  |
| bio18 | -0.896 | 0.040 | -0.217 | 0.543 | -0.808 | -0.957 | 0.364 | -0.791 | -0.795 | -0.878 | -0.926 | 0.936 | 0.557 | 0.988 | -0.944 | 0.629 | 0.993 | 1.000 |  |
| bio19 | -0.605 | -0.315 | -0.246 | -0.232 | -0.672 | -0.497 | -0.311 | -0.754 | -0.496 | -0.649 | -0.523 | 0.720 | 0.663 | 0.478 | -0.240 | 0.854 | 0.454 | 0.496 | 1.000 |
