# Supplementary material for: Phylogeographic Insights into a Peripheral Refugium: The Importance of Cumulative Effect of Glaciation on the Genetic Structure of Two Endemic Plants
Source: PLoS One. 2016 Nov 21;11(11):e0166983. doi: 10.1371/journal.pone.0166983 (PMC5117763; doi:10.1371/journal.pone.0166983)
Supplement: S2 Table — (DOCX) [file pone.0166983.s002.docx]

**S1 TABLE.** Number of models replication, number of pseudoabsences and methods used for selecting pseudoabsences for each SDM technique. Selecion of parameters followed the recommendation of Babet-Massin *et al.* (2012).

| **Model** | **Number of models replication** | **number of pseudoabsences** | **Selection method** |
| --- | --- | --- | --- |
| MARS | 20 | 100 | random |
| GLM | 1 | 10000 | random |
| CTA | 10 | equal to number of occurrences | sre |
| FDA | 20 | 100 | sre |
| RF | 10 | equal to number of occurrences | sre |
| MAX | 10 | 10000 | random |
