# Supplementary material for: Phylogeographic Insights into a Peripheral Refugium: The Importance of Cumulative Effect of Glaciation on the Genetic Structure of Two Endemic Plants
Source: PLoS One. 2016 Nov 21;11(11):e0166983. doi: 10.1371/journal.pone.0166983 (PMC5117763; doi:10.1371/journal.pone.0166983)
Supplement: S3 Table — (DOCX) [file pone.0166983.s003.docx]

**S2 TABLE.** Primers description. (F) forward and (R) reverse, (length) length of primer, (Ta) Temperature of annealing and (Te) temperature of extension in *Silene cordifolia* and *Viola* *argenteria*.

|  |  |  | ***S. cordifolia*** | | ***V. argenteria*** | |  |
| --- | --- | --- | --- | --- | --- | --- | --- |
| **Primer name** | **F/R** | **length** | **Ta** | **Te** | **Ta** | **Te** | **Sequence** |
| *5'trnG2G* | F | 23 | 49 | 72 | 48 | 72 | GCGGGTATAGTTTAGTGGTAAAA |
| *3'trnG^UUC^* | R | 23 |  |  |  |  | GTAGCGGGAATCGAACCCGCATC |
| *trnH^GUG^* | F | 20 | 55 | 72 | 50 | 72 | ACTGCCTTGATCCACTTGGC |
| *psbA* | R | 21 |  |  |  |  | CGAAGCTCCATCTACAAATGG |
| *rpoC1F* | F | 21 |  |  | 50 | 72 | GGATACACTTCTTGATAATGG |
| *rpoC1R* | R | 21 |  |  |  |  | TGAGAAAACATAAGTAAACGG |
| *atpB* | F | 21 |  |  | 50 | 72 | ACTCGCACACACTCCCTTTCC |
| *atpH* | R | 23 |  |  |  |  | GCTTTTATGGAAGCTTTAACAAT |
| *trnT_a_* | F | 20 | 63 | 72 | 51 | 72 | CATTACAAATGCGATGCTCT |
| *trnL_b_* | R | 20 |  |  |  |  | TCTACCGATTTCGCCATATC |
| *trnL_c_* | F | 20 |  |  | 52 | 72 | CGAAATCGGTAGACGCTACG |
| *trnL_d_* | R | 20 |  |  |  |  | GGGGATAGAGGGACTTGAAC |
| *rps12* | F | 23 |  |  | 54 | 72 | ATTAGAAANRCAAGACAGCCAAT |
| *rpl20* | R | 20 |  |  |  |  | CGYYAYCGAGCTATATATCC |
| *trnC^GCA^F* | F | 18 | 53.5 | 72 |  |  | CCAGTTCRAATCYGGGTG |
| *ycf6R* | R | 26 |  |  |  |  | GCCCAAGCRAGACTTACTATATCCAT |
| *rps16F* | F | 20 | 52 | 72 |  |  | AAACGATGTGGTARAAAGCAA |
| *rps16R* | R | 24 |  |  |  |  | AACATCWATTGCAASGATTCGATA |
| *trnQ^UUG^* | F | 20 | 56 | 72 |  |  | GCGTGGCCAAGYGGTAAGGC |
| *5'rps16* | R | 22 |  |  |  |  | GTTGCTTTYTACCACATCGTTT |
| *trnL_c_* | F | 20 | 60 | 72 |  |  | CGAAATCGGTAGACGCTACG |
| *trnF_f_* | R | 19 |  |  |  |  | ATTTGAACTGGTGACACGA |
