# Supplementary material for: Phylogeographic Insights into a Peripheral Refugium: The Importance of Cumulative Effect of Glaciation on the Genetic Structure of Two Endemic Plants
Source: PLoS One. 2016 Nov 21;11(11):e0166983. doi: 10.1371/journal.pone.0166983 (PMC5117763; doi:10.1371/journal.pone.0166983)
Supplement: S4 Table — (DOCX) [file pone.0166983.s004.docx]

**S3 TABLE**. Sampled populations of *Silene cordifolia* and *Viola argenteria*, with GenBank accession numbers of plastid sequences (atpF-atpH, rpoC1 gene, rps12-rpL20, rps16 gene, trnG2G-trnG, trnH-psbA , trnLc-trnLd, trnC-ycf6R, trnL-trnF , trnQ-rps16, trnTa-trnLb).

| *Silene cordifolia* | rps16 gene | trnC-ycf6 | trnL-trnF | trnQ-rps16 | trnS-trnG | trnT-trnL | trnH-psbA |
| --- | --- | --- | --- | --- | --- | --- | --- |
| Pop1 | KU608353  KU608354  KU608355  KU608356  KU608357 | KU608447  KU608448  KU608449  KU608450  KU608451 | KU608541  KU608542  KU608543  KU608544  KU608545 | KU608635  KU608636  KU608637  KU608638  KU608639 | KU608729  KU608730  KU608731  KU608732  KU608733 | KU608823  KU608824  KU608825  KU608826  KU608827 | KU608917  KU608918  KU608919  KU608920  KU608921 |
| Pop2 | KU608358  KU608359  KU608360  KU608361  KU608362 | KU608452  KU608453  KU608454  KU608455  KU608456 | KU608546  KU608547  KU608548  KU608549  KU608550 | KU608640  KU608641  KU608642  KU608643  KU608644 | KU608734  KU608735  KU608736  KU608737  KU608738 | KU608828  KU608829  KU608830  KU608831  KU608832 | KU608922  KU608923  KU608924  KU608925  KU608926 |
| Pop3 | KU608363  KU608364  KU608365  KU608366  KU608367 | KU608457  KU608458  KU608459  KU608460  KU608461 | KU608551  KU608552  KU608553  KU608554  KU608555 | KU608645  KU608646  KU608647  KU608648  KU608649 | KU608739  KU608740  KU608741  KU608742  KU608743 | KU608833  KU608834  KU608835  KU608836  KU608837 | KU608927  KU608928  KU608929  KU608930  KU608931 |
| Pop4 | KU608368  KU608369  KU608370  KU608371  KU608372 | KU608462  KU608463  KU608464  KU608465  KU608466 | KU608556  KU608557  KU608558  KU608559  KU608560 | KU608650  KU608651  KU608652  KU608653  KU608654 | KU608744  KU608745  KU608746  KU608747  KU608748 | KU608838  KU608839  KU608840  KU608841  KU608842 | KU608932  KU608933  KU608934  KU608935  KU608936 |
| Pop5 | KU608373  KU608374  KU608375  KU608376  KU608377 | KU608467  KU608468  KU608469  KU608470  KU608471 | KU608561  KU608562  KU608563  KU608564  KU608565 | KU608655  KU608656  KU608657  KU608658  KU608659 | KU608749  KU608750  KU608751  KU608752  KU608753 | KU608843  KU608844  KU608845  KU608846  KU608847 | KU608937  KU608938  KU608939  KU608940  KU608941 |
| Pop6 | KU608378  KU608379  KU608380  KU608381  KU608382 | KU608472  KU608473  KU608474  KU608475  KU608476 | KU608566  KU608567  KU608568  KU608569  KU608570 | KU608660  KU608661  KU608662  KU608663  KU608664 | KU608754  KU608755  KU608756  KU608757  KU608758 | KU608848  KU608849  KU608850  KU608851  KU608852 | KU608942  KU608943  KU608944  KU608945  KU608946 |
| Pop7 | KU608383  KU608384  KU608385  KU608386  KU608387 | KU608477  KU608478  KU608479  KU608480  KU608481 | KU608571  KU608572  KU608573  KU608574  KU608575 | KU608665  KU608666  KU608667  KU608668  KU608669 | KU608759  KU608760  KU608761  KU608762  KU608763 | KU608853  KU608854  KU608855  KU608856  KU608857 | KU608947  KU608948  KU608949  KU608950  KU608951 |
| Pop8 | KU608388  KU608389  KU608390  KU608391  KU608392 | KU608482  KU608483  KU608484  KU608485  KU608486 | KU608576  KU608577  KU608578  KU608579  KU608580 | KU608670  KU608671  KU608672  KU608673  KU608674 | KU608764  KU608765  KU608766  KU608767  KU608768 | KU608858  KU608859  KU608860  KU608861  KU608862 | KU608952  KU608953  KU608954  KU608955  KU608956 |
| Pop9 | KU608393  KU608394  KU608395  KU608396 | KU608487  KU608488  KU608489  KU608490 | KU608581  KU608582  KU608583  KU608584 | KU608675  KU608676  KU608677  KU608678 | KU608769  KU608770  KU608771  KU608772 | KU608863  KU608864  KU608865  KU608866 | KU608957  KU608958  KU608959  KU608960 |
| Pop10 | KU608397  KU608398  KU608399  KU608400 | KU608491  KU608492  KU608493  KU608494 | KU608585  KU608586  KU608587  KU608588 | KU608679  KU608680  KU608681  KU608682 | KU608773  KU608774  KU608775  KU608776 | KU608867  KU608868  KU608869  KU608870 | KU608961  KU608962  KU608963  KU608964 |
| Pop11 | KU608401  KU608402  KU608403  KU608404  KU608405 | KU608495  KU608496  KU608497  KU608498  KU608499 | KU608589  KU608590  KU608591  KU608592  KU608593 | KU608683  KU608684  KU608685  KU608686  KU608687 | KU608777  KU608778  KU608779  KU608780  KU608781 | KU608871  KU608872  KU608873  KU608874  KU608875 | KU608965  KU608966  KU608967  KU608968  KU608969 |
| Pop12 | KU608406  KU608407  KU608408  KU608409  KU608410  KU608411 | KU608500  KU608501  KU608502  KU608503  KU608504  KU608505 | KU608594  KU608595  KU608596  KU608597  KU608598  KU608599 | KU608688  KU608689  KU608690  KU608691  KU608692  KU608693 | KU608782  KU608783  KU608784  KU608785  KU608786  KU608787 | KU608876  KU608877  KU608878  KU608879  KU608880  KU608881 | KU608970  KU608971  KU608972  KU608973  KU608974  KU608975 |
| Pop13 | KU608412  KU608413  KU608414  KU608415  KU608416 | KU608506  KU608507  KU608508  KU608509  KU608510 | KU608600  KU608601  KU608602  KU608603  KU608604 | KU608694  KU608695  KU608696  KU608697  KU608698 | KU608788  KU608789  KU608790  KU608791  KU608792 | KU608882  KU608883  KU608884  KU608885  KU608886 | KU608976  KU608977  KU608978  KU608979  KU608980 |
| Pop14 | KU608417  KU608418  KU608419  KU608420  KU608421 | KU608511  KU608512  KU608513  KU608514  KU608515 | KU608605  KU608606  KU608607  KU608608  KU608609 | KU608699  KU608700  KU608701  KU608702  KU608703 | KU608793  KU608794  KU608795  KU608796  KU608797 | KU608887  KU608888  KU608889  KU608890  KU608891 | KU608981  KU608982  KU608983  KU608984  KU608985 |
| Pop15 | KU608422  KU608423  KU608424  KU608425  KU608426 | KU608516  KU608517  KU608518  KU608519  KU608520 | KU608610  KU608611  KU608612  KU608613  KU608614 | KU608704  KU608705  KU608706  KU608707  KU608708 | KU608798  KU608799  KU608800  KU608801  KU608802 | KU608892  KU608893  KU608894  KU608895  KU608896 | KU608986  KU608987  KU608988  KU608989  KU608990 |
| Pop16 | KU608427  KU608428  KU608429  KU608430  KU608431 | KU608521  KU608522  KU608523  KU608524  KU608525 | KU608615  KU608616  KU608617  KU608618  KU608619 | KU608709  KU608710  KU608711  KU608712  KU608713 | KU608803  KU608804  KU608805  KU608806  KU608807 | KU608897  KU608898  KU608899  KU608900  KU608901 | KU608991  KU608992  KU608993  KU608994  KU608995 |
| Pop17 | KU608432  KU608433  KU608434  KU608435  KU608436 | KU608526  KU608527  KU608528  KU608529  KU608530 | KU608620  KU608621  KU608622  KU608623  KU608624 | KU608714  KU608715  KU608716  KU608717  KU608718 | KU608808  KU608809  KU608810  KU608811  KU608812 | KU608902  KU608903  KU608904  KU608905  KU608906 | KU608996  KU608997  KU608998  KU608999  KU609000 |
| Pop18 | KU608437  KU608438  KU608439  KU608440  KU608441 | KU608531  KU608532  KU608533  KU608534  KU608535 | KU608625  KU608626  KU608627  KU608628  KU608629 | KU608719  KU608720  KU608721  KU608722  KU608723 | KU608813  KU608814  KU608815  KU608816  KU608817 | KU608907  KU608908  KU608909  KU608910  KU608911 | KU609001  KU609002  KU609003  KU609004  KU609005 |
| Pop19 | KU608442  KU608443  KU608444  KU608445  KU608446 | KU608536  KU608537  KU608538  KU608539  KU608540 | KU608630  KU608631  KU608632  KU608633  KU608634 | KU608724  KU608725  KU608726  KU608727  KU608728 | KU608818  KU608819  KU608820  KU608821  KU608822 | KU608912  KU608913  KU608914  KU608915  KU608916 | KU609006  KU609007  KU609008  KU609009  KU609010 |
|  |  |  |  |  |  |  |  |
| *Viola argenteria* | atpF-atpH | rpoC1 gene | rps12-rpl20 | trnH-psbA | TrnLc-trnLd | trnS-trnG | TrnT-trnF |
| Pop01 | KU558128  KU558129  KU558130  KU558131 | KU558208  KU558209  KU558210  KU558211 | KU558288  KU558289  KU558290  KU558291 | KU558368  KU558369  KU558370  KU558371 | KU558448  KU558449  KU558450  KU558451 | KU558528  KU558529  KU558530  KU558531 | KU558608  KU558609  KU558610  KU558611 |
| Pop02 | KU558132  KU558133  KU558134  KU558135 | KU558212  KU558213  KU558214  KU558215 | KU558292  KU558293  KU558294  KU558295 | KU558372  KU558373  KU558374  KU558375 | KU558452  KU558453  KU558454  KU558455 | KU558532  KU558533  KU558534  KU558535 | KU558612  KU558613  KU558614  KU558615 |
| Pop03 | KU558136  KU558137  KU558138  KU558139  KU558140 | KU558216  KU558217  KU558218  KU558219  KU558220 | KU558296  KU558297  KU558298  KU558299  KU558300 | KU558376  KU558377  KU558378  KU558379  KU558380 | KU558456  KU558457  KU558458  KU558459  KU558460 | KU558536  KU558537  KU558538  KU558539  KU558540 | KU558616  KU558617  KU558618  KU558619  KU558620 |
| Pop04 | KU558141  KU558142  KU558143  KU558144  KU558145 | KU558221  KU558222  KU558223  KU558224  KU558225 | KU558301  KU558302  KU558303  KU558304  KU558305 | KU558381  KU558382  KU558383  KU558384  KU558385 | KU558461  KU558462  KU558463  KU558464  KU558465 | KU558541  KU558542  KU558543  KU558544  KU558545 | KU558621  KU558622  KU558623  KU558624  KU558625 |
| Pop05 | KU558146  KU558147  KU558148  KU558149  KU558150  KU558151 | KU558226  KU558227  KU558228  KU558229  KU558230  KU558231 | KU558306  KU558307  KU558308  KU558309  KU558310  KU558311 | KU558386  KU558387  KU558388  KU558389  KU558390  KU558391 | KU558466  KU558467  KU558468  KU558469  KU558470  KU558471 | KU558546  KU558547  KU558548  KU558549  KU558550  KU558551 | KU558626  KU558627  KU558628  KU558629  KU558630  KU558631 |
| Pop06 | KU558152  KU558153  KU558154  KU558155  KU558156 | KU558232  KU558233  KU558234  KU558235  KU558236 | KU558312  KU558313  KU558314  KU558315  KU558316 | KU558392  KU558393  KU558394  KU558395  KU558396 | KU558472  KU558473  KU558474  KU558475  KU558476 | KU558552  KU558553  KU558554  KU558555  KU558556 | KU558632  KU558633  KU558634  KU558635  KU558636 |
| Pop07 | KU558157  KU558158  KU558159  KU558160  KU558161 | KU558237  KU558238  KU558239  KU558240  KU558241 | KU558317  KU558318  KU558319  KU558320  KU558321 | KU558397  KU558398  KU558399  KU558400  KU558401 | KU558477  KU558478  KU558479  KU558480  KU558481 | KU558557  KU558558  KU558559  KU558560  KU558561 | KU558637  KU558638  KU558639  KU558640  KU558641 |
| Pop08 | KU558162  KU558163  KU558164 | KU558242  KU558243  KU558244 | KU558322  KU558323  KU558324 | KU558402  KU558403  KU558404 | KU558482  KU558483  KU558484 | KU558562  KU558563  KU558564 | KU558642  KU558643  KU558644 |
| Pop09 | KU558200  KU558201 | KU558280  KU558281 | KU558360  KU558361 | KU558440  KU558441 | KU558520  KU558521 | KU558600  KU558601 | KU558680  KU558681 |
| Pop10 | KU558165  KU558166  KU558167 | KU558245  KU558246  KU558247 | KU558325  KU558326  KU558327 | KU558405  KU558406  KU558407 | KU558485  KU558486  KU558487 | KU558565  KU558566  KU558567 | KU558645  KU558646  KU558647 |
| Pop11 | KU558168  KU558169  KU558170  KU558171  KU558172 | KU558248  KU558249  KU558250  KU558251  KU558252 | KU558328  KU558329  KU558330  KU558331  KU558332 | KU558408  KU558409  KU558410  KU558411  KU558412 | KU558488  KU558489  KU558490  KU558491  KU558492 | KU558568  KU558569  KU558570  KU558571  KU558572 | KU558648  KU558649  KU558650  KU558651  KU558652 |
| Pop12 | KU558173  KU558174  KU558175  KU558176 | KU558253  KU558254  KU558255  KU558256 | KU558333  KU558334  KU558335  KU558336 | KU558413  KU558414  KU558415  KU558416 | KU558493  KU558494  KU558495  KU558496 | KU558573  KU558574  KU558575  KU558576 | KU558653  KU558654  KU558655  KU558656 |
| Pop13 | KU558177  KU558178  KU558179  KU558180  KU558181 | KU558257  KU558258  KU558259  KU558260  KU558261 | KU558337  KU558338  KU558339  KU558340  KU558341 | KU558417  KU558418  KU558419  KU558420  KU558421 | KU558497  KU558498  KU558499  KU558500  KU558501 | KU558577  KU558578  KU558579  KU558580  KU558581 | KU558657  KU558658  KU558659  KU558660  KU558661 |
| Pop14 | KU558182  KU558183  KU558184 | KU558262  KU558263  KU558264 | KU558342  KU558343  KU558344 | KU558422  KU558423  KU558424 | KU558502  KU558503  KU558504 | KU558582  KU558583  KU558584 | KU558662  KU558663  KU558664 |
| Pop15 | KU558185  KU558186  KU558187  KU558188  KU558189 | KU558265  KU558266  KU558267  KU558268  KU558269 | KU558345  KU558346  KU558347  KU558348  KU558349 | KU558425  KU558426  KU558427  KU558428  KU558429 | KU558505  KU558506  KU558507  KU558508  KU558509 | KU558585  KU558586  KU558587  KU558588  KU558589 | KU558665  KU558666  KU558667  KU558668  KU558669 |
| Pop16 | KU558190  KU558191  KU558192 | KU558270  KU558271  KU558272 | KU558350  KU558351  KU558352 | KU558430  KU558431  KU558432 | KU558510  KU558511  KU558512 | KU558590  KU558591  KU558592 | KU558670  KU558671  KU558672 |
| Pop17 | KU558193  KU558194  KU558195  KU558196 | KU558273  KU558274  KU558275  KU558276 | KU558353  KU558354  KU558355  KU558356 | KU558433  KU558434  KU558435  KU558436 | KU558513  KU558514  KU558515  KU558516 | KU558593  KU558594  KU558595  KU558596 | KU558673  KU558674  KU558675  KU558676 |
| Pop18 | KU558197  KU558198  KU558199 | KU558277  KU558278  KU558279 | KU558357  KU558358  KU558359 | KU558437  KU558438  KU558439 | KU558517  KU558518  KU558519 | KU558597  KU558598  KU558599 | KU558677  KU558678  KU558679 |
| Pop19 | KU558202  KU558203 | KU558282  KU558283 | KU558362  KU558363 | KU558442  KU558443 | KU558522  KU558523 | KU558602  KU558603 | KU558682  KU558683 |
| Pop20 | KU558204  KU558205 | KU558284  KU558285 | KU558364  KU558365 | KU558444  KU558445 | KU558524  KU558525 | KU558604  KU558605 | KU558684  KU558685 |
| Pop21 | KU558206  KU558207 | KU558286  KU558287 | KU558366  KU558367 | KU558446  KU558447 | KU558526  KU558527 | KU558606  KU558607 | KU558686  KU558687 |
