# Supplementary material for: Phylogeographic Insights into a Peripheral Refugium: The Importance of Cumulative Effect of Glaciation on the Genetic Structure of Two Endemic Plants
Source: PLoS One. 2016 Nov 21;11(11):e0166983. doi: 10.1371/journal.pone.0166983 (PMC5117763; doi:10.1371/journal.pone.0166983)
Supplement: S5 Table — (DOCX) [file pone.0166983.s005.docx]

**S4 TABLE**. Phylogeographic utility of seven plastid DNA sequence loci of *Silene cordifolia* and of seven plastid DNA sequence loci of *Viola argenteria* screened for the molecular phylogeographic study.

| **Species** | **Region** | **Length** | **Polimorphic sites** | **Parsimony informative sites** | **Indels** |
| --- | --- | --- | --- | --- | --- |
| ***S. cordifolia*** |  |  |  |  |  |
|  | *rps16* gene | 813 | 0 | 0 | 0 |
|  | *trnG2G-trnG* | 678 | 2 | 2 | 0 |
|  | *trnH-psbA* | 332 | 2 | 2 | 0 |
|  | trnL-*trnF* | 860 | 2 | 2 | 0 |
|  | *trnT_a_-trnL_b_* | 624 | 6 | 3 | 1 |
|  | *trnQ-rps16* | 702 | 0 | 0 | 0 |
|  | *trnC-ycf6R* | 452 | 0 | 0 | 0 |
| ***V. argenteria*** |  |  |  |  |  |
|  | *atpF-atpH* | 703 | 4 | 4 | 1 |
|  | *rpoC1* gene | 474 | 0 | 0 | 0 |
|  | *rps12-rpL20* | 752 | 2 | 2 | 7 |
|  | *trnG2G-trnG* | 642 | 1 | 1 | 4 |
|  | *trnH-psbA* | 387 | 8 | 8 | 5 |
|  | *trnL-trnF* | 459 | 0 | 0 | 0 |
|  | *trnT_a_-trnL_b_* | 313 | 0 | 0 | 0 |
