# Supplementary material for: Phylogeographic Insights into a Peripheral Refugium: The Importance of Cumulative Effect of Glaciation on the Genetic Structure of Two Endemic Plants
Source: PLoS One. 2016 Nov 21;11(11):e0166983. doi: 10.1371/journal.pone.0166983 (PMC5117763; doi:10.1371/journal.pone.0166983)

**S1 FIG**. Maps showing the consensus among the 6 binary maps of the Last Glacial Maximum distribution (LGM ~ 21 kya) for *S. cordifolia* (A and B) and *V. argenteria* (C and D). Models were obtained using six algorithms (MARS, GLM, CTA, FDA, RF and MAX). LGM models are obtained using three palaeoclimate data (CCSM, MIROC and MPI). The inferred continuous probability values were converted to binary using TSS threshold. Red indicates greatest consensus among models and blue indicates lowest consensus among models. Current known distribution area of *S. cordifolia* (continuous black line) and *V. argenteria* (dotted black line) is reported.


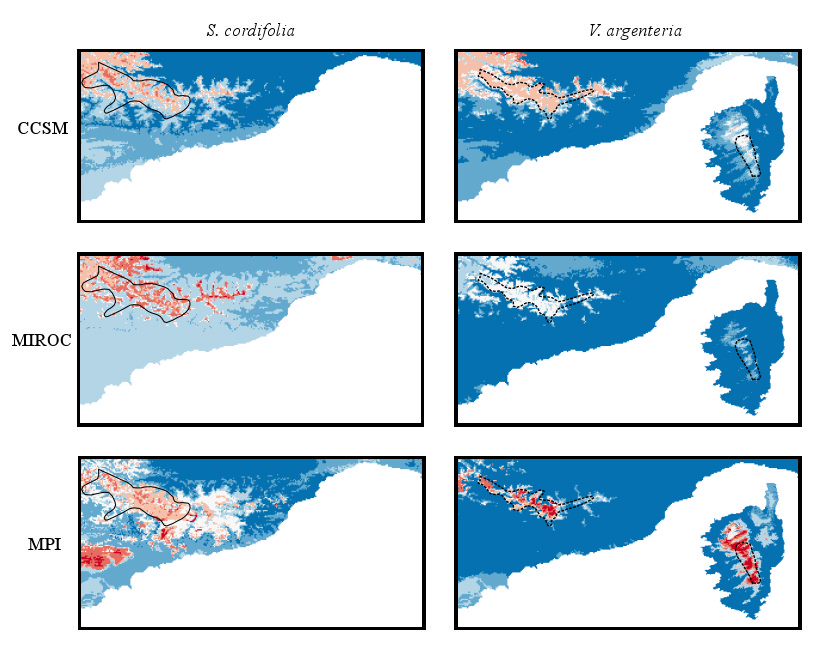

Supplement: S1 Fig — (DOCX) [file pone.0166983.s006.docx]
