# Supplementary material for: Phylogeographic Insights into a Peripheral Refugium: The Importance of Cumulative Effect of Glaciation on the Genetic Structure of Two Endemic Plants
Source: PLoS One. 2016 Nov 21;11(11):e0166983. doi: 10.1371/journal.pone.0166983 (PMC5117763; doi:10.1371/journal.pone.0166983)

**S2 FIG**. Results of the spatial analyses of molecular variance (SAMOVA) in *Silene cordifolia* (a) and *Viola argenteria* (b) populations. Fct and Fsc values obtained when searching for K=2 to 10 groups using cpDNA sequences.


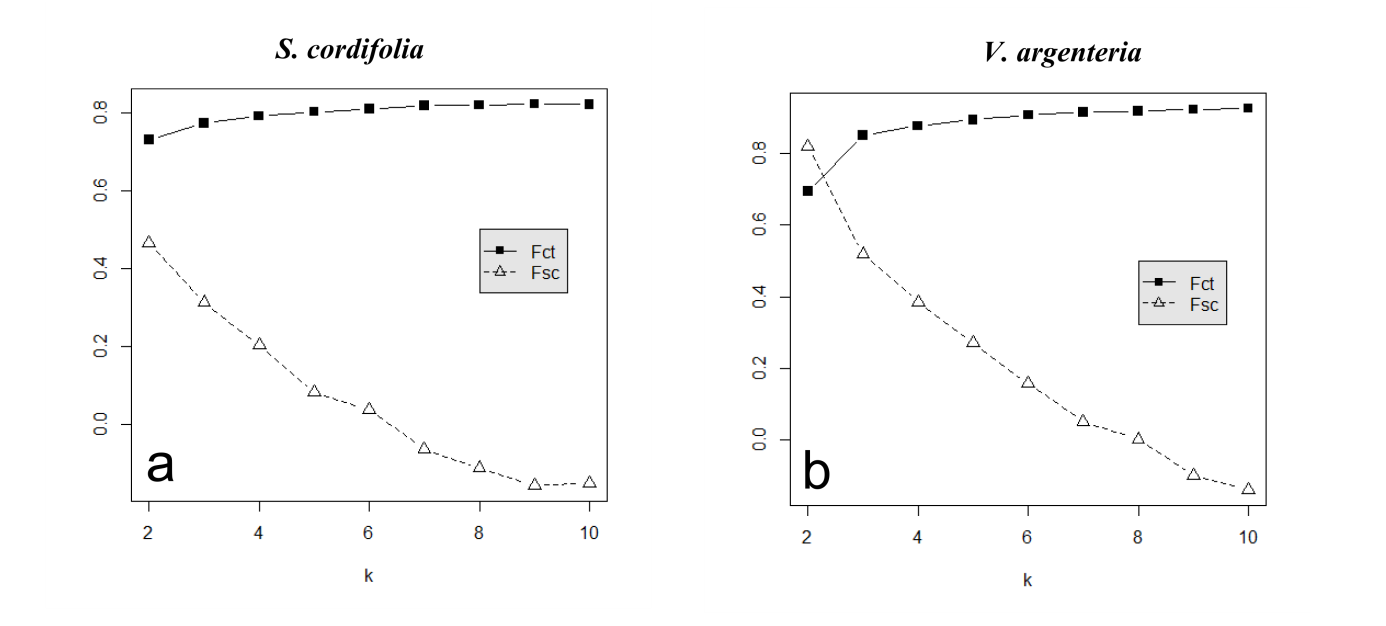

Supplement: S2 Fig — (DOCX) [file pone.0166983.s007.docx]
